# Loop Diuretic Administration in Patients with Acute Heart Failure and Reduced Systolic Function: Effects of Different Intravenous Diuretic Doses and Diuretic Response Measurements

**DOI:** 10.3390/jcm8111854

**Published:** 2019-11-02

**Authors:** Gaetano Ruocco, Mauro Feola, Ranuccio Nuti, Lorenzo Luschi, Isabella Evangelista, Alberto Palazzuoli

**Affiliations:** 1 Department of Internal Medicine, Cardiovascular Disease Unit, Le Scotte Hospital, University of Siena, 53100 Siena, Italy; gmruocco@virgilio.it (G.R.); nutir@unisi.it (R.N.); lorenzo.luschi89@gmail.com (L.L.); isabella.evangelista87@gmail.com (I.E.); 2 Cardiology Section, Regina Montis Regalis Hospital, Mondovì, 12084 Cuneo, Italy; mauro.feola@aslcn1.it

**Keywords:** acute heart failure, outcome, loop diuretics, diuretic response, congestion

## Abstract

Background: Despite the fact that loop diuretics are a landmark in acute heart failure (AHF) treatment, few trials exist that evaluate whether the duration and timing of their administration and drug amount affect outcome. In this study, we sought to evaluate different loop diuretic infusion doses in relation to outcome and to diuretic response (DR), which was serially measured during hospitalization. Methods: This is a post-hoc analysis of a DIUR-HF trial. We divided our sample on the basis of intravenous diuretic dose during hospitalization. Patients taking less than 125 mg of intravenous furosemide (median value) were included in the low dose group (LD), patients with a diuretic amount above this threshold were inserted in the high dose group (HD). The DR formula was defined as weight loss/40 mg daily of furosemide and it was measured during the first 24 h, 72 h, and over the whole infusion period. Outcome was considered as death due to cardiovascular causes or heart failure hospitalization. Results: One hundred and twenty-one AHF patients with reduced ejection fractions (EF) were evaluated. The cardiovascular (CV) death/heart failure (HF) re-hospitalization rate was significantly higher in the HD group compared to the LD group (75% vs. 22%; *p* < 0.001). Both low DR, measured during the entire infusion period (HR 3.25 (CI: 1.92–5.50); *p* < 0.001) and the intravenous diuretic HD (HR 5.43 [CI: 2.82–10.45]; *p* < 0.001) were related to outcome occurrence. Multivariable analysis showed that DR (HR 3.01 (1.36–6.65); *p* = 0.006), intravenous diuretic HD (HR 2.83 (1.24–6.42); *p*=0.01) and worsening renal function (WRF) (HR 2.21 (1.14–4.28); *p* = 0.01) were related to poor prognosis. Conclusions: HD intravenous loop diuretic administration is associated with poor prognosis and less DR. Low DR measured during the whole intravenous administration better predicts outcome compared to DR measured in the early phases. ClinicalTrials.gov Acronym and Identifier Number: DIUR-HF; NCT01441245; registered on 23 September 2011.

## 1. Introduction

Loop diuretics are the most commonly used drug in acute heart failure (AHF) treatment, but their short and long-term effects are relatively unknown. Nevertheless, high quality data supporting the optimal infusion amount, the appropriate dosage after intravenous (IV) administration, and timing of the infusion are lacking [1,2]. Recommended guidelines focus primarily on symptom relief since there are no specific studies evaluating the dose regimen or modality of administration during acute management [3]. Thus, despite the ubiquity of loop diuretic usage, there is a lack of data providing information on the prognostic impact in relation to dose administration and infusion modality. This is the only randomized study that has evaluated both the doses (high versus low) as well as the modality of administration (infusion versus bolus) arms [4]. Conversely, results from the Evaluation Study of the Congestive Heart Failure and Pulmonary Artery Catheterization Effectiveness (ESCAPE) trial showed that independent of the diuretic response, high diuretic doses during hospitalization are associated with increased mortality at 6-months [5]. Similarly, the Acute Decompensated Heart Failure National Registry (ADHERE) found that high dosages of loop diuretics were associated with significant mortality risk [6]. Overall, it is not clear what the optimal intravenous amount should be in order to avoid diuretic resistance. Notably, several formulas have recently been proposed in order to target the optimal dose. Both responsiveness metrics are based on weight loss or diuresis per 40 mg of furosemide infusion or by urine sample measurement [7,8,9,10,11].

To date, there are no prospective interventional studies on the relationship between loop diuretic modality administration and diuretic efficiency. To clarify these concerns, we evaluated different intravenous loop diuretic amounts (high vs. low dose) in relation to the occurrence of residual congestion, diuretic response (DR) and outcome. We also evaluated the prognostic significance of DR measured over the total intravenous furosemide administration in comparison with DR measured during the early hospitalization period.

## 2. Methods

### 2.1. Study Design

This is a post-hoc analysis of a randomized, open label study comparing continuous (cIV) with intermittent (iIV) furosemide infusion in patients admitted with a diagnosis of AHF and a left ventricular ejection fraction (LVEF) <50% in our tertiary-care medical center. Echocardiography was performed within 48 h of hospital admission. Patients were enrolled consecutively from the Department of Internal Medicine, Cardiology Section Center (Siena, Italy) from September 2013 to January 2017. The modalities of furosemide administration, in hospital treatment and patients’ eligibility were described in our previous study [12]. We divided our sample on the basis of the intravenous diuretic dose during hospitalization. Patients taking less than 125 mg of intravenous furosemide (median value) were considered the low lose group (LD).

Patients taking thiazide diuretics, nesiritide, or arginine vasopressin antagonists were excluded. Therefore, subjects with end-stage renal disease or the need for renal replacement therapy (dialysis or ultrafiltration), isolated diastolic dysfunction with LVEF ≥50% or recent myocardial infarction within thirty days were excluded. This trial was registered in ClinicalTrials.gov on 27 September 2011 with the identifier number: *NCT01441245*, and was updated regularly. The study was conducted in accordance with the Helsinki Declaration criteria, all patients gave written consent and the study was previously approved by our institutional review board (C.E.A.V.S.E.).

### 2.2. Definitions

Chronic kidney disease (CKD) was defined as an estimated glomerular filtration rate (eGFR) <60 mL/min/1.73 m^2^ at baseline. Worsening renal function (WRF) was defined as a serum creatinine increase of ≥0.3 mg/dL or an eGFR decrease of ≥20% at any time from admission to discharge [13]. eGFR was calculated using the modification of diet in renal disease (MDRD) equation [14]. A BNP cut off >100 pg /mL at baseline, which is associated with the typical signs and symptoms of AHF and a positive radiological chest X-ray were considered the criteria for patients’ inclusion in the study. A congestion score that gives one point for each clinical sign of volume overload was measured both at admission and at discharge [15]. The following congestion signs were evaluated: pulmonary rales, third heart sound, jugular vein distension, peripheral edema and hepatomegaly (5 total points).

Diuretic response (DR) was defined as daily weight loss per 40 mg of furosemide and was examined at day 1, day 3 and during the whole infusion period. Low DR was defined on the basis of the values above the median. We also collected the daily in-hospital dosage of furosemide.

### 2.3. Follow-Up

Clinical outcome was evaluated in terms of death for cardiovascular causes or heart failure hospitalization over a 6-month follow-up period. There was a scheduled outpatient visit or phone contact at 30, 60, 90 and 180 days after discharge. Measurements of outcome were done during both the in-hospital and post discharge period. Heart failure hospitalization was considered as any hospital admission with a primary or secondary diagnosis of volume overload or low output due to pump failure, acute coronary syndrome complicated by heart failure, ventricular arrhythmia associated with left ventricular dysfunction, or heart failure related to WRF. All these events were defined through our results as a composite outcome in terms of HF re-hospitalization/cardiovascular (CV) mortality. The in-hospital events related to diuretic administration such as electrolytes unbalance and/or hypotension were defined as loop diuretic side effects.

### 2.4. Laboratory Analysis

A complete blood analysis including hemoglobin concentration, hematocrit, red blood cell count, serum creatinine, sodium, and potassium was performed at the time of admission to determine the baseline criteria, and subsequent testing was performed each day and again at the time of discharge. Plasma B-type natriuretic peptide (BNP) was measured at baseline and at the end of the infusion period using an immune-fluorescence assay manufactured by Inverness (San Diego, CA, USA).

### 2.5. Statistical Analysis

All data were analyzed with intention-to-treat principles. Continuous variables were expressed as a median (interquartile range (IQR)), while discrete variables were presented as counts with percentages (%). The Mann-Whitney *U* test and χ^2^-test were used as indicated to compare groups. Differences between the diuretic response quartiles were compared by the analysis of variance when distributed approximately normally or by using the Kruskal-Wallis test when skewed. The χ^2^ test was used to compare categorical data. The receiving operating characteristics (ROC) curve was employed to evaluate the outcome prediction by oral loop diuretics dosage at discharge. The Cox regression analysis was used to assess the independent relationship between the DR measurement for the outcome of rehospitalization or death with adjustment for age, gender, cardiovascular risk factors (hypertension, smoking, dyslipidemia, diabetes mellitus, coronary artery disease), decrease in BNP, atrial fibrillation (confirmed by the presence of this arrhythmia on ECG examination or monitoring during hospitalization), the use of hyperosmolar solution and dopamine infusion. This adjustment was based on previous studies on this topic; we did not adjust this analysis according to LVEF because in this study, we included only patients with reduced LVEF (LVEF < 50%). Moreover, we did not include the background therapy of patients in the multivariable model as there was no difference between patients in the HD and LD subgroups. The Kaplan-Meier method was employed to generate survival plots, using the log-rank test, for the composite outcome. All statistical tests were two-tailed, with *p*-value <0.05 considered significant. All the analyses were performed by using the SPSS 20.0 for Windows (SPSS, IBM).

## 3. Results

**Sample**: A total of 138 patients consecutively admitted with AHF were evaluated: six were excluded because of a diagnosis of HF with preserved ejection fraction, four affected by severe CKD and two due to in-hospital sudden death. Of 126 patients, five patients were excluded because of the lack of complete clinical and laboratory data. Median age was 81 (76–86) years, the median baseline creatinine value was 1.50 (1.20–1.80) mg/dL and the median baseline BNP was 842 (545–1289) pg/mL. Fifty-one patients (42%) were male, and 57 patients (47%) experienced CKD at admission. About one quarter of the population (34 patients) experienced WRF during treatment. The overall median urine output was 2100 (1700–25000 ml/day and the median furosemide daily intravenous (IV) dose was 125 (IQR 100–200) mg/die. The median DR during the whole period of furosemide infusion was 0.20 (0.091–0.266).

**Admission clinical and laboratory characteristics according to diuretic dose:** Patients who received a high IV loop diuretic dose (n. 71) demonstrated, at admission, a significantly higher rate of congestion score >2 compared to the LD group (63% vs. 36%; *p* = 0.003), as well as higher serum levels of admission creatinine (1.70 (1.20–2.10) vs. 1.35 (1.17–1.59) mg/dL; *p* = 0.005), higher serum levels of blood urea nitrogen (BUN) (85 (59–112) vs. 66 (42–106) mg/dL; *p* = 0.04), higher serum levels of potassium (4.2 (4.0–4.5) vs. 4.0 (3.7–4.5) mEq/l; *p* = 0.02), lower eGFR (41 (32–5)] vs. 46 (38–58) ml/min/1.73 m2; *p* = 0.04), lower serum levels of sodium (138 (130–142) vs. 140 (138–143) mEq/l; *p* = 0.007) and lower median LVEF (30 (25–35)% vs. 40 (35–40)%; *p* < 0.001) compared to patients in the LD group. Moreover, patients receiving HD were more likely to be afflicted with diabetes mellitus (51% vs. 22%; *p* = 0.001) and baseline CKD (61% vs. 28%; *p* < 0.001). No differences between groups were found in terms of background therapy (Table 1).

**Discharge clinical and laboratory characteristics according to diuretic dose:** We evaluated laboratory and clinical parameters after treatment, and found that HD patients had significantly higher serum levels of discharge creatinine (1.60 (1.30–2.20) vs. 1.40 (1.16–1.60) mg/dL; *p* = 0.006), lower eGFR (40 (33–51) vs. 47 (42–56) ml/min/1.73 m2; *p* = 0.008) and lower LVEF (30 (25–35)% vs. 40 (35–40)%; *p* < 0.001) compared to the LD group; no differences were found between the two groups in terms of admission BNP levels, WRF development and persistence of congestion at discharge. DR response at day 1 was not significantly different between the HD and LD groups. However, DR at day 3 (0.106 (0.053–0.213) vs. 0.222 (0.127–0.407); *p* < 0.001) and DR during the entire infusion period (0.106 (0.064–0.240) vs. 0.266 (0.200–0.400); *p* < 0.001) were lower in the HD group with respect to the LD group. The outcome events rate was significantly higher (75% vs. 22%; *p* < 0.001) in HD patients compared with those receiving LD of IV furosemide infusion (Table 2).

**Clinical and laboratory characteristics in relation to DR quartiles:** Dividing the entire DR into quartiles, patients with impaired DR (group 1 and 2) showed, as statistically significant, lower discharge eGFR values (36 (30–51) vs. 43 (27–50) vs. 49 (44–51) vs. 47 (38–54); *p* = 0.01), lower median LVEF (30 (25–35) vs. 30 (25–35) vs. 35 (30–40) vs. 40 (35–40); *p* < 0.001) and higher intravenous diuretic dosage during hospitalization (200 (175–225) vs. 200 (125–250) vs. 120 (80–125) vs. 100 (71–125); *p* < 0.001). Therefore, subgroups with lower DR (1st and 2nd quartiles), demonstrated a significant higher rate of outcome occurrence (71% vs. 73% vs. 37% vs. 34%; *p* = 0.001) with respect to subgroups in higher quartiles of (3rd and 4th quartiles) (Table 3).

**Figure 1:**
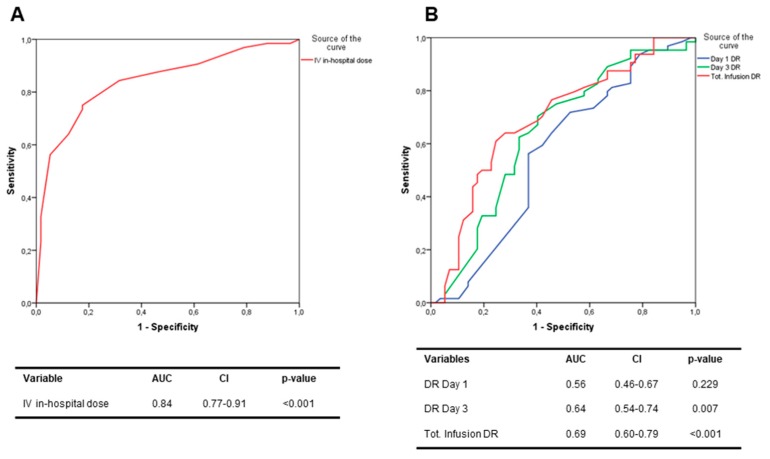
Receiving operating characteristics (ROC) curve analysis for 180 days outcome prediction of IV furosemide dosage (**A**); ROC curve analysis for 180 days outcome prediction of lower DR measured at day 1, at day 3 and during whole IV infusion (**B**).

**Outcome analysis:** Our sample showed 64 events after 180 days of follow-up, which included 20 deaths and 44 re-hospitalizations. ROC curve analysis showed that the amount of the HD infusion diuretic was able to predict poor prognosis (AUC 0.84 (CI 0.77–0.91); *p* < 0.001). (Figure 1A) DR was measured at day 1, day 3 and during the whole period of furosemide infusion. ROC curve analysis demonstrated that increased DR measured during the entire infusion period (AUC 0.69 (CI 0.60–0.79); *p* < 0.001) proved to be the best prognostic tool compared to DR measured at day 3 (AUC 0.64 (CI 0.54–0.74); *p* = 0.007) and DR measured at day 1 (AUC 0.56 (CI 0.46–0.67); *p* = 0.229), which was not statistically significant (Figure 1B).

**Figure 2:**
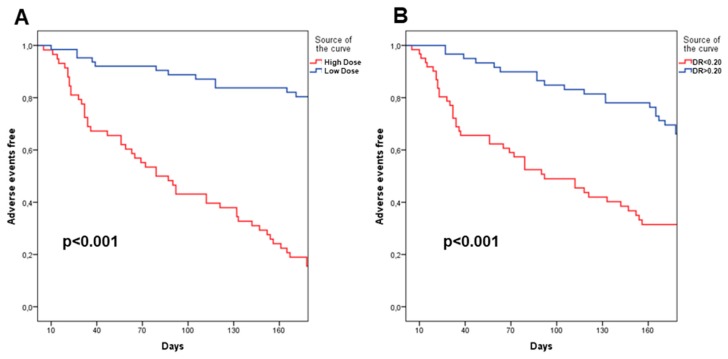
Kaplan Meier 180 days survival curves in patients divided into high vs. low IV furosemide doses (**A**); Kaplan Meier 180 days survival curves in patients divided into high vs. low DR measurements during entire infusion period (**B**).

Univariate Cox regression analysis demonstrated that either DR measured during the entire infusion period (HR 3.25 (CI: 1.92–5.50); *p* < 0.001) and IV HD diuretic dose (HR 5.43 (CI: 2.82–10.45); *p* < 0.001) were related to outcome occurrence. After adjustment for potential confounders, a multivariable analysis showed that DR measured during the whole infusion (HR 3.01 (1.36–6.65); *p* = 0.006), IV HD diuretic dose (HR 2.83 (1.24–6.42); *p* = 0.01) and WRF (HR 2.21 (1.14–4.28); *p* = 0.01) were related to poor prognosis (Table 4). Kaplan Meier survival curves confirmed the prognostic role of DR measured during the whole infusion and of the HD of oral furosemide at discharge (Figure 2A,B).

## 4. Discussion

The present study demonstrated that patients with higher intravenous diuretic dosage displayed an increased risk for outcome occurrence compared to patients taking a lower dose. The current findings suggest that the prognostic impact of high infusion administration has a close relationship with adverse outcome during the 6-month follow-up period. The HD amount also appears to be related to worse DR, which in turn, is associated with poor outcome. Interestingly, patients taking a higher amount showed impaired clinical conditions including congestion, and kidney and cardiac systolic function. Moreover, the HD group showed an increased need for additional treatment during intravenous diuretic infusion. From this current analysis, we can assume that elevated diuretic dosage is necessary to solve congestion in patients with more advanced HF and worsened clinical conditions. Indeed, the admission congestion score, renal function parameters and BUN values were higher in the HD group, whereas BNP levels and DR measured during the early hospitalization period were similar in both the HD and LD groups. Moreover, the inverse relation existing between HD and low DR suggests that this specific group deserves additional diuretic treatment that stresses the nephron in different sites in order to obtain more efficient diuresis. The current data differs from the DOSE trial as it did not show any statistical difference between groups receiving high vs. low doses of diuretics. [16] Perhaps, this is due to the benefits of an aggressive decongestion strategy, which was potentially nullified by an equivalent magnitude of adverse effects due to the high loop diuretic dose. Secondly, it could be due to the mean older age and worse clinical conditions of our sample compared to the DOSE patients. Notably, looking at our patients’ characteristics, we can observe a more elevated diuretic dose before randomization together with a reduced percentage of betablocker and anti-aldosterone drug. Therefore, the mean BUN and eGFR were significantly higher in our sample. Unfortunately, the clinical congestion and natriuretic peptide levels are not comparable because we applied a different congestion score and BNP measurement rather than NTproBNP. However, considering all these features, we can argue that our sample differs significantly from the DOSE patients. In this respect, Hanberg et al. in a post-hoc analysis of DOSE, demonstrated that a more aggressive loop diuretic strategy is of potential benefit, but when adjusted for diuretic dose and degree of decongestion, the potential benefits disappear [17]. The inverse relationship existing between diuretic dose and DR, deserves specific research and to be evaluated in relation to outcome. Notably, several trials have demonstrated that patients taking an elevated diuretic dose have an impaired outcome compared to those taking a low amount: high doses are associated with higher rates of CKD, advanced NYHA class, and congestion severity [18,19]. Other analyses have suggested that higher doses of diuretics are necessary in severe cases and that adverse effects may result from disease severity [20,21,22]. Similarly, in a recent study, patients taking diuretics had a worse clinical and laboratory pattern and this was related to an impaired prognosis [23]. Conversely, in the propensity matched analysis of the Acute Heart Failure Global Registry of Standard Treatment (ALARM-HF) study, bolus and high dosage administration did not influence short-term mortality [24]. Thus, both poor DR and HD amount could depend on the disease severity or other systemic disorders, evaluated by a comprehensive staging of multi-organ damage [25].

Another important item that needs to be highlighted is the prognostic relevance of DR measured during the entire infusion period, which appears to be a more useful tool for risk stratification with respect to traditional analysis, which evaluates DR only after early intravenous administration. In theory, our proposed analysis which assesses the whole infusion time frame, better reflects systemic congestion and renal modifications. Indeed, DR formulas have been recently proposed to identify the diuretic response during the infusion period most based on the natriuretic response, and a urine sample soon after the first loop diuretic administration [7,10,11]. This offers several advantages for diuresis monitoring, the diuretic dose suitability, and rapid titration in order to avoid breaking phenomena. Despite these advantages, some aspects such as the glomerular filtration, tubular function, the fluid balance management and the effective decongestion during the whole infusion period have not been precisely evaluated [26,27,28]. Our findings indicate that some subjects, those with an acceptable response during the first days, can develop some degree of diuretic resistance after the first early infusion and they deserve persistent diuresis monitoring. Recently, a similar proposed formula has been described by Aoki et al., although congestion was evaluated only by the presence of peripheral edema and jugular vein distention [29]. Nevertheless, the findings substantially reflect our data. Up to now, a universal protocol or modality to guide the use of diuretics has not been standardized and loop diuretics have been administered based on proper clinical practice rather than on a precise protocol [30]. We believe that the measurement of DR across the infusion period, could help physicians to target the oral pre-discharge dosage and customize the risk for adverse event occurrence.

### Limitations

This is a post-hoc analysis of a single controlled study, and it is prone to several forms of bias due to the nature of the protocol and intervention. Firstly, this is a smaller study with respect to other AHF studies. However, the mean dose during step-by-step administration was similar in both arms during the protocol treatment. There are some biases in the protocol study due to the diuretic dosage before randomization, the titration of the furosemide dose according to the patient’s response and non-uniform standard forms of therapy (i.e., nitrate ACE-inhibitors, beta-blockers). Results could be partially influenced by the diuretic dose regimen during the first 12 h before randomization. Most of the patients received open-label diuretic therapy during the period before randomization and before hospital admission. Our findings cannot be extended to patients with preserved systolic function taking lower diuretic requirements. To this end, we are still enrolling patients and organizing a multi-center study including either reduced or preserved systolic function patients [31]. The equation that as applied is an extension of a previously validated formula for infusion timing, however, it needs external validation in a larger cohort. An additional analysis evaluating the urine sodium level and its relationship with the current formula and outcome prediction should be performed. Finally, the study provided a standardized protocol dose and modality administration, and although it was not randomized with a control group, the current results could be applicable in a vulnerable population with advanced age, taking high oral diuretic doses.

## 5. Conclusions

Although an elevated amount of diuretic is necessary to achieve optimal decongestion in AHF patients, the high infusion diuretic dose is related to adverse outcomes. This trend could be attributed to a greater disease severity and lower DR. The DR measured during the entire infusion period appears to better predict outcome than the DR measured during early hospitalization. The identification of a specific pattern including DR measured over the entire diuretic infusion and intravenous diuretic amount during hospitalization, could be of potential interest for a better risk assessment after AHF hospitalization. A precise algorithm comprising the measurement of diuretic dose, clinical congestion score, and DR could optimize the oral diuretic titration before discharge, and thus reduce the hospitalization rate. The prognostic accuracy of contemporary evaluation of the current parameters would be warranted by a prospective randomized study.

## Figures and Tables

**Table 1:** Differences in clinical and laboratory characteristics at admission among patients receiving HD vs. patients receiving LD of intravenous furosemide

	HD (*n* = 71)	LD (*n* = 50)	*p*-Value
**Age (years)**	82 (77–87)	79 (76–85)	0.13
**Gender (*n*)**			
Male	30	21	0.98
Female	41	29	(Ref)
**Laboratory data at admission**			
Creatinine (mg/dL)	1.70 (1.20–2.10)	1.35 [1.17–1.59)	0.005
eGFR (ml/min/1.73 m2)	41 (32–51)	46 [38–58)	0.04
BUN (mg/dL)	85 (59–112)	66 [42–106)	0.04
Serum sodium (mEq/L)	138 (130–142)	140 [138–143)	0.007
Serum potassium (mEq/L)	4.2 (4.0–4.5)	4.0 (3.7–4.5)	0.02
BNP (pg/mL)	887 (563–1350)	837 (530–1275)	0.68
**LVEF (%)**	30 (25–35)	40 (35–40)	<0.001
**Admission congestion score >2 (%)**	63	36	0.003
**Risk Factors (%)**			
Diabetes	51	22	0.001
Dyslipidemia	49	44	0.57
Hypertension	48	44	0.67
CAD	69	54	0.09
CKD	61	28	<0.001
AF	39	30	0.29
**Background therapy (%)**			
Loop diuretics	100	96	0.33
ACE/ARBs	59	64	0.73
Beta blockers	72	76	0.76
Aldosterone antagonists	24	22	0.98

Abbreviations: atrial fibrillation (AF); B-type natriuretic peptide (BNP); blood urea nitrogen (BUN); chronic kidney disease (CKD); coronary artery disease (CAD); estimated glomerular filtration rate (eGFR); high dose (HD); left ventricular ejection fraction (LVEF); low dose (LD).

**Table 2:** Differences in clinical and laboratory characteristics at discharge among patients receiving HD vs. patients receiving LD of intravenous furosemide.

	HD (*n* = 71)	LD (*n* = 50)	*p*-Value
**Laboratory data after treatment**			
Creatinine (mg/dL)	1.60 (1.30–2.20)	1.40 (1.16–1.50)	0.006
eGFR (ml/min/1.73 m2)	40 (33–51)	47 (42–56)	0.008
Serum sodium (mEq/L)	138 (135–142)	138 (136–140)	0.81
Serum potassium (mEq/L)	4.0 (3.8–4.4)	3.8 (3.6–4.3)	0.28
BNP (pg/mL)	620 (381–824)	610 (378–928)	0.79
Urine output (ml/die)	2200 (1700–2700)	2000 (1800–2500)	0.57
**Additional hypertonic saline solution (%)**	41	20	0.02
**Inotropes agents administration (%)**	34	14	0.01
**WRF (%)**	29	28	0.98
**Persistence of congestion after treatment (%)**	25	34	0.30
**180 days outcome events occurrence (%)**	75	22	<0.001
**DR day 1**	0.20 (0.00–0.32)	0.33 (0.00–0.64)	0.17
**DR day 2**	0.106 (0.053–0.213)	0.222 (0.127–0.407)	<0.001
**DR entire infusion period**	0.106 (0.064–0.240)	0.266 (0.200–0.400)	<0.001

Abbreviations: B-type natriuretic peptide (BNP); diuretic response (DR); estimated glomerular filtration rate (eGFR); high dose (HD); low dose (LD).

**Table 3:** Differences in clinical and laboratory characteristics among quartiles of DR (median value: 0.20 (0.091–0.266).

	Q1 (*n* = 31)	Q2 (*n* = 26)	Q3 (*n* = 35)	Q4 (*n* = 29)	*p*-Value
**Age (years)**	81 (76–87)	83 (76–88)	81 (78–85)	79 (77–85)	0.76
**Gender male (%)**	32	42	40	55	0.34
**Renal function biomarkers at admission**					
Creatinine (mg/dL)	1.70 (1.10–1.80)	1.70 (1.27–2.12)	1.40 (1.20–1.80)	1.50 (1.10–1.70)	0.33
eGFR (ml/min/1.73 m2)	39 (32–58)	41 (24–50)	45 (38–52)	49 (38–60)	0.15
BUN (mg/dL)	79 (56–104)	87 (59–105)	67 (42–106)	73 (48–117)	0.68
**Renal function biomarkers after treatment**					
Creatinine (mg/dL)	1.50 (1.40–1.90)	1.55 (1.27–2.30)	1.40 (1.20–1.60)	1.50 (1.20–1.80)	0.17
eGFR (ml/min/1.73 m2)	36 (30–51)	43 (27–50)	49 (44–51)	47 (38–54)	0.01
**Admission BNP (pg/mL)**	753 (536–1214)	861 (563–1436)	875 (585–1270)	887 (452–1401)	0.97
**Mean daily urine output (ml)**	1900 (1500–2250)	2550 (1850–3100)	2100 (1700–2500)	2400 (1850–2800)	0.01
**Diuretic in-hospital IV dosage (mg/die)**	200 (175–225)	200 (125–250)	120 (80–125)	100 (71–125)	<0.001
**LVEF (%)**	30 (25–35)	30 (25–35)	35 (30–40)	40 (35–40)	<0.001
**Additional hypertonic saline solution (%)**	26	54	23	31	0.06
**Inotropes agents administration (%)**	16	42	20	28	0.12
**Risk factors (%)**					
Diabetes Mellitus	71	27	23	34	<0.001
Hypertension	48	65	31	45	0.07
Dyslipidemia	48	77	40	28	0.002
CAD	87	58	43	66	0.003
**Atrial fibrillation (%)**	65	35	11	34	<0.001
**CKD (%)**	58	58	34	41	0.15
**BNP decrease ≥ 30% after treatment (%)**	48	54	49	48	0.97
**WRF (%)**	26	19	23	45	0.13
**Persistence of congestion after treatment (%)**	26	23	40	24	0.39
**180 days CV death/HF rehospitalization (%)**	71	73	37	34	0.001

Abbreviations: atrial fibrillation (AF); B-type natriuretic peptide (BNP); blood urea nitrogen (BUN); chronic kidney disease (CKD); coronary artery disease (CAD); diuretic response (DR); estimated glomerular filtration rate (eGFR); left ventricular ejection fraction (LVEF).

**Table 4:** Cox regression analysis for outcome prediction at 180 days. Abbreviations: B-type natriuretic peptide (BNP); confidence interval (CI); diuretic response (DR); high dose (HD); hazard ratio (HR); worsening renal function (WRF).

DEATH or RE-HOSPITALIZATION (180 Days)				
	Univariate		Multivariable	
*Parameters*	*HR (95% CI of HR)*	*p-Value*	*HR ^1^ (95% CI of HR)*	*p-Value*
low DR	3.25 (1.92–5.50)	<0.001	3.01 (1.36–6.65)	0.006
HD IV dosage furosemide	5.43 (2.82–10.45)	<0.001	2.83 (1.24–6.42)	0.01
WRF	1.17 (0.68–1.99)	0.573	2.21 (1.14–4.28)	0.01
Persistence of congestion *	0.81 (0.47–1.42)	0.466	0.55 (0.27–1.13)	0.10

* At discharge; ^1^ Adjusted for age, gender, risk factors, atrial fibrillation, BNP decrease, hypertonic saline solution administration and inotropes infusion.

## Abbreviations

ADHERE	Acute Decompensated Heart Failure National Registry
AHF	Acute heart failure
ALARM-HF	Acute Heart Failure Global Registry of Standard Treatment
BNP	B-type natriuretic peptide
cIV	Continuous intravenous infusion
CKD	Chronic kidney disease
DOSE	Diuretic strategies in acute decompensated heart failure
DR	Diuretic response
eGFR	Estimated glomerular filtration rate;
ESCAPE	Evaluation Study of Congestive Heart Failure and Pulmonary Artery Catheterization Effectiveness
HD	High dose
HF	Heart failure
iIV	Intermittent intravenous infusion
IQR	Interquartile range
IV	Intravenous
LD	Low dose
MDRD	Modification of diet in renal disease
PROTECT	Placebo-controlled randomized study of the selective A(1) adenosine receptor antagonist rolofylline
RELAX-AHF	Relaxin in acute heart failure
ROC	Receiving operating characteristics
WRF	Worsening renal function
